# Prevalence of diabetes and unrecognized diabetes in hypertensive patients aged 40 to 79 years in southwest China

**DOI:** 10.1371/journal.pone.0170250

**Published:** 2017-02-13

**Authors:** Xiao-bo Huang, Wei-wei Tang, Ya Liu, Rong Hu, Ling-yun Ouyang, Jian-xiong Liu, Xiu-jun Li, Yan-jing Yi, Tzung-Dau Wang, Shui-ping Zhao

**Affiliations:** 1 Department of Cardiology, Cent S Univ, Xiangya Hosp 2, Changsha, Hunan, China; 2 Department of Cardiology, the second people’s hospital of Chengdu, Chengdu, Sichuan Province, China; 3 West China School of Public Health, Sichuan University, Chengdu, Sichuan Province, China; 4 Division of Cardiology, The Second Affiliated Hospital of Chongqing Medical University, Chongqing, China; 5 Department of Endocrinology and Metabolism, the second people’s hospital of Chengdu, Chengdu, Sichuan Province, China; 6 Department of Endocrinology and Metabolism, West China Hospital of Sichuan University, Chengdu, People’s Republic of China; 7 Division of Cardiology, Department of Internal Medicine, National Taiwan University Hospital, Taipei City, Taiwan; University of Catanzaro, ITALY

## Abstract

This study aimed to assess the prevalence of diabetes and unrecognized diabetes in hypertensive patients aged 40 to 79 years in Southwest China. From September 2013 to March 2014, a cross-sectional survey was conducted in 4021 hypertensive patients aged 40 to 79 years living in Chengdu and Chongqing, China. Fasting plasma glucose (FPG) and 2h plasma glucose (2-hPG) in an oral glucose-tolerance test (OGTT) were used for assessments. Whether the patients previously had diabetes (DM) was determined by their own reports. The survey was carried out by the same questionnaire for all respondents. DM prevalence was 32.0% in hypertensive patients aged 40 to 79 years in Southwest China, with the rates of 29.6% and 33.5% in men and women, respectively (*P*<0.001). DM prevalence increased with age age and body-mass index. DM prevalence rates were 16.9%, 24.7%, 38.2% and 41.9% in hypertensive patients aged 40–49, 50–59, 60–69 and over 70, respectively. DM prevalence were 30.6%, 27.9%, 37.1%, and 37.4%, for BMI<18.5, 18.5–24.9, 25.0–29.9, and ≥30, respectively. Prevalence of unrecognized DM were 20.8% in hypertensive patients aged 40 to 79 years in Southwest China. Using only fasting blood glucose testing without OGTT would have resulted in 65.0% of missed DM diagnosis in these newly diagnosed patients. The prevalence of DM and unrecognized DM were high in hypertensive patients aged 40 to 79 years in Southwest China.These findings indicate that hypertensive patients aged 40 to 79 years should regularly submit to community-based OGTT screening for timely DM diagnosis.

## Introduction

Hypertension is a common chronic disease, and a major risk factor for cardiovascular diseases. It greatly increases the risk of stroke, myocardial infarction, heart failure, and chronic kidney disease[1]. In China, the prevalence of hypertension in adults over 20 years of age is 26.6%, with about 250 million individuals suffering from high blood pressure [2]. As another risk factor for cardiovascular diseases, DM is highly prevalent in China. Indeed, 9.7% of adults over 20 years have DM, i.e. 92 million Chinese [3]. Cardiovascular disease prevention and control faces huge challenges. If hypertensive patients also have DM, the occurrence and development of atherosclerosis might be accelerated. Specifically, the risk of cardiovascular diseases in hypertensive patients with DM is at least twice than patients only suffering from hypertension [4]. In addition, it is harder to control blood pressure in hypertensive patients with DM than those without DM. Three antihypertensive drugs are often needed for hypertensive patients with DM [5]. Thus, early diagnosis and intervention of DM in hypertensive patients are of great significance.

In China, epidemiological studies assessing the prevalence of DM in hypertensive population are scarce. Qin et al reported the prevalence of DM, based on fasting plasma glucose (FPG) alone, in hypertensive patients aged 45–75 years was 13.2% in Lianyungang, a city in eastern China [6]. Obviously, this number may not reflect the true prevalence of DM in hypertensive patients because 2-h plasma glucose (2-hPG) in an oral glucose tolerance test (OGTT) was not carried out. Hence, diabetic patients only showing postprandial hyperglycemia were left unrecognized. We therefore carried out the present epidemiological survey to assess hypertensive patients aged 40–79 years in the urban areas of Chongqing and Chengdu in southwest China. FPG combined with OGTT 2-hPG were used to assess patients’ glucose status. This analysis of the epidemiological status of DM in hypertensive individuals provides a knowledge base for better implementation of prevention and control of cardiovascular diseases in China, the largest developing country.

## Methods

### Study subjects

This was a cross-sectional survey conducted in hypertensive individuals. This survey was conducted in urban communities of Chongqing and Chengdu, using a multi-stage stratified sampling method. In the first phase, Jinjiang, Longquan and Chenghua districts were randomly selected in the urban area of Chengdu; Yubei and Jiangbei districts were randomly selected in Chongqing. In the second phase, a random sub-district was selected in each district. In the third stage, one community was randomly selected in each sub-district, total of five random communities were selected.

### Selection criteria

Inclusion criteria for the study were: (1) more than 5 years of residence in the community; (2) 40–79 years old; (3) systolic blood pressure (SBP)≥140 mmHg and /or diastolic blood pressure (DBP)≥90 mmHg, and /or diagnosis of hypertension and currently under antihypertensive treatment. Exclusion criteria were mental illness, renal insufficiency requiring dialysis, and end-stage cancer. Patients declining study participation were also excluded. Two blood pressure readings were obtained on the right arm with the participant in a seated position after 5 minutes of rest, and averaged. Blood pressure was measured with a regular mercury sphygmomanometer. The first (appearance) and fifth (disappearance) Korotkoff sounds were considered to indicate SBP and DBP, respectively. Based on the above inclusion and exclusion criteria, 4836 hypertensive patients were enrolled, of which 4478 participated in the study from September 2013 to March 2014. Due to missing demographic information or blood glucose data, 457 patients were excluded. Thus, 4021 patients were included in the final analysis.

### Data collection

More than 30 investigators were trained for data collection. All subjects filled the same on-site questionnaire, which included demographic characteristics, lifestyle risk factors, personal and family history, according to the cardiovascular survey methods of WHO[7]. The questionnaire also included height, weight, waist circumference, and blood pressure. When measuring the height and weight, the subject needed to be barefoot and take off any hat, wearing only lightweight clothes. Body-mass index (BMI) was calculated as weight (kg) by height (in meters) squared.

### Blood sample collection and laboratory tests

Venous blood was drawn after 12 hours fasting. Patients without a history of DM were submitted to OGTT: 75 g of glucose dissolved in 300 ml of warm water was administered orally and within five minutes, venous blood was drawn 2 hour later (2-hPG). All blood samples were sent to the Clinical Laboratory Center of Second People's Hospital of Chengdu and Clinical Laboratory Center of the Second Affiliated Hospital of Chongqing Medical University. Both laboratories are up-to-date with the national standards. Blood glucose, lipids and uric acid were assessed in all blood samples. Total cholesterol(TC), triglyceride (TG), and blood glucose amounts were detected by enzymatic method. High-density lipoprotein cholesterol(HDL-C) and low density lipoprotein cholesterol(LDL-C) levels were measured using a homogeneous method. Serum uric acid was measured by the phosphotungstic acid method on an automatic biochemical analyzer.

### Diagnosis standards

According to the US JNC-7 standard, high blood pressure was defined as SBP ≥140 mmHg and/or DBP ≥90 mmHg, and/or being diagnosed with hypertension and currently under antihypertensive drug treatment [8]. DM was defined as FPG level ≥7.0 mmol/L, 2-hPG level ≥11.1 mmol/L, or with a previous clinical diagnosis [9]. Overweight was defined as a body-mass index between 25.0 and 29.9, Obesity was defined as a body-mass index of 30.0 or more [10]. Central obesity was defined as a waist circumference of 90 cm or more in men and as 80 cm or more in women [11]. Hypertriglyceridemia was defined as TG level ≥1.7 mmol/L based on the criteria of the NCEP Adult Treatment Panel III report [12]. History of smoking was defined as smoking at least once per day over a year, and having smoked or quitted smoking less than three years ago.History of drinking was considered if drinking at least once a week over a year, and having drank or quitted drinking less than three years ago. Family history of hypertension was defined as immediate family members (grandparents, parents, or sibling) having hypertension. Family history of DM was defined as immediate family members (grandparents, parents, or sibling) having DM. Physical exercise was defined as having at least one exercise session per week.

### Sample size estimation

There are a lack of data for prevalence of DM among Chinese hypertensive patients of communities, thus, we calculated sample size based on prevalence of 18% for DM in the population aged 40–79 years in chengdu (china) 2008 [13]. If the prevalence of DM were 18%, the estimated sample size was 2893, the actual sample size in the study was 4021.

### Statistical analysis

The Epidata3.1 software was used to double input data and ensure their quality. Data processing and analysis were carried out with the SAS9.2 software (Institute Inc. SAS, Cary, NC, USA,). Qualitative data were compared by χ^2^ test, and quantitative data by one sample t-test or Wilcoxon rank sum test. The χ^2^ linear trend test was used to detect the trend of DM prevalence in middle-aged and older hypertensive individuals, in association with age and BMI. The logistic regression was used to explore the potential risk factor. P<0.05 was treated as significance.

## Results

### Baseline characteristics of subjects

**Fig 1:**
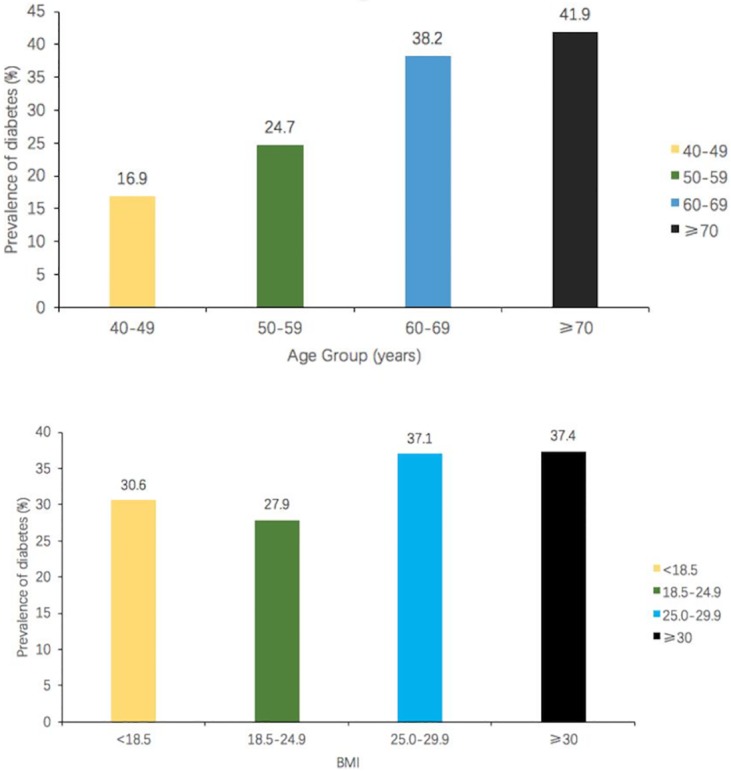
DM prevalence in hypertensive patients aged 40 to 79 years gradually increased with age and BMI.

This study totally included 4021 Chinese hypertensive individuals, of 40 to 79 (62.2 ± 29.3) years old. Among them, 79.6% had high school education or less, and 80.3% had 2,000 yuan (US $ 314.0) or less as monthly income. Of the 4021 hypertensive patients, 1286 (32.0%) had DM. The prevalence of DM was 29.6% in men and 33.5% in women (*P*<0.001). Interestingly, DM prevalence in hypertensive individuals gradually increased with age and BMI (*P*<0.001). (Fig 1).

Of the 1286 (32.0%) diabetic patients, 449 (11.2%) had been previously diagnosed, and 837 (20.8%) were newly diagnosed. In other words, the prevalence of unrecognized DM in hypertensive patients aged 40 to 79 years in Southwest China were 20.8%. Unrecognized DM cases accounted for 65.1% (837/1286) of all patients with DM. Among patients less than 50 years old, 89.5% of diabetic patients were newly diagnosed. The ratio of newly diagnosed DM cases decreased with age (*P* value for linear trend <0.01). For patients over 70, the ratio of newly diagnosed DM cases was 55.4%. (Table 1)

**Table 1:** Diabetes detection in hypertensive populations.

Parameter	Previous diagnosed	Newly diagnosed	Proportion of newly diagnosed*
N (%)	449(11.2%)	837(20.8%)	837(65.1%)
Gender			
Male	165(10.7%)	293(18.9%)	64.0%
Female	284(11.5%)	544(22.0%)	65.7%
Age group			
<50	8(1.8%)	68(15.1%)	89.5%
50~59	90(6.6%)	248(18.1%)	73.4%
60~69	189(14.2%)	320(24.0%)	62.9%
≥70	162(18.7%)	201(23.2%)	55.4%

*Linear trend *P*<0.01

Among the newly diagnosed DM patients, only 35.0% would have been diagnosed if only fasting glucose levels were measured. 36.4% and 32.4% in females and males, respectively. These findings indicated that DM diagnosis rates were low (about 29.4–42.7% for all age groups) when fasting glucose tests only were carried out. In newly DM diagnosed cases, 86.7% patients would have been found if only 2-hPG was used. Consequently, undiagnosed rate was relatively low using the latter assay. In both sex and age groups, DM diagnosis rates were high (all above 80 percent), using only 2-hPG for detection (Table 2).

**Table 2:** Detection rates of newly diagnosed diabetes in hypertensive populations using two different methods.

	(newly diagnosed, N)	Using FPG	Using 2-hpG
Total	837	293 (35.0%)	726(86.7%)
Gender			
Male	293	95 (32.4%)	248(84.6%)
Female	544	198 (36.4%)	478(82.9%)
Age group			
<50	68	27(39.7%)	61(89.7%)
50~59	248	106(42.7%)	207(83.5%)
60~69	320	109(34.1%)	286(89.4%)
≥70	201	51(25.4%)	172(85.6%)

### Univariate analyses

Hypertensive men had higher weight, waist circumferences and uric acid level compared with hypertensive women. Meanwhile, females had higher BMI, TC, HDL-C, LDL-C, FPG, and 2-hPG compared with male. No significant differences were observed in systolic blood pressure, diastolic blood pressure, heart rate, TG and family history of DM were obtained between men and women (Table 3).

**Table 3:** Baseline characteristics of the hypertensive population.

Groups	Overall(n = 4021)	Male(n = 1549)	Female(n = 2472)	*P* values
Age, mean(SD)	62.2(29.3)	62.9(29.8)	61.7(29.0)	0.200
Weight, kg, mean (SD)	61.6(16.8)	66.0(17.0)	58.9(16.1)	<0.001
BMI, kg/m2, mean (SD)	25.1(8.0)	24.7(7.1)	25.3(8.6)	0.010
Never smoking(%)	2981(74.6)	624(40.6)	2357(95.8)	<0.001
Never drinking(%)	3188(80.0)	850(55.7)	2338(95.2)	<0.001
Education lever lower than high school(%)	3178(79.6)	1030(66.8)	2148(87.5)	<0.001
Income less than 2000 yuan(%)	3174(80.3)	1117(73.8)	2057(84.4)	<0.001
systolic pressure, mmHg, mean (SD)	150.9(18.7)	150.7(18.3)	151(18.9)	0.590
Diastolic pressure/mmHg, mean (SD)	88.6(25.9)	89.3(18.2)	88.1(29.8)	0.110
Heart rate/min, mean (SD)	82.1(29.7)	81.8(32.6)	82.24(27.7)	0.680
TC, mmol/L, mean (SD)	4.7(0.9)	4.6(0.9)	4.8(0.9)	<0.001
HDL-C, mmol/L, mean (SD)	1.4(0.3)	1.3(0.3)	1.4(0.3)	<0.001
LDL-C, mmol/L, mean (SD)	2.6(0.8)	2.5(0.8)	2.6(0.8)	<0.001
TG, mmol/L, mean (SD)	1.8(1.4)	1.8(1.4)	1.8(1.4)	0.180
FPG, mmol/l, mean (SD)	6.0(2.1)	5.9(1.9)	6.0(2.3)	0.010
2hPG, mmol/L, mean (SD)	8.8(4.0)	8.5(3.8)	9.0(4.2)	<0.001
uric acid, mmol/L, mean (SD)	310.5(90.0)	359.2(90.9)	280.4(74.2)	<0.001
Family history of diabetes %	344(8.6)	135(8.7)	209(8.5)	0.765
waist circumference, cm, mean (SD)	85.5(16.2)	87.6(25.2)	84.2(10.0)	0.001

### Multiple logistic regression

We conducted a logistic regression to confirm some potential risk factors. Diabetes status was treated as a dependent variable, and other factors including age,sex, History of smoking, History of drinking, lack of exercise, family history of hypertension, overweight or obesity, central obesity, and TG, family history of diabetes and so on were treated as independent variable. The logistics regression results found that age, lack of physical exercised and family history of hypertension, overweight or obesity, central obesity and high TG level and family history of DM are associated with diabetes. The specific results were shown in Table 4.

**Table 4:** Logistics regression for DM among the hypertensive populations.

Variable	Odds Ratios (95% CI)	*P* Value
Age, per 10-yr increment	1.33(1.16~1.53)	<0.001
lack of physical exercises	1.32(1.17~1.48)	<0.001
Family history of hypertension	1.61(1.26~2.06)	0.004
overweight or obesity	1.28(0.98~1.68)	0.005
Central obesity	1.32(1.00~1.73)	0.040
hypertriglyceridemia	1.59(1.18~2.15)	0.002
Family history of DM	1.83(1.06~3.16)	0.040

## Discussion

From 2013 to 2014, a cross-sectional survey was carried out in hypertensive populations aged 40 to 79 years in two cities of China, Chengdu and Chongqing, and the investigation found DM prevalence was 32.0%. These findings suggested that approximately one-in-three hypertensive patients aged 40 to 79 years are diabetics. The DM prevalence in Chinese hypertensive patients aged 40 to 79 years was higher than that obtained in Spain (25.4%) from 420 patients over 18 with essential hypertension [14]. This discrepancy might be attributed to higher average age of the current study patients; indeed, this prevalence is much higher than that of the general Chinese population (9.7%) [3]. A higher DM prevalence for women aged 40 to 79 years was obtained in comparison with male counterparts, suggesting that women with hypertension more easily develop into DM. With increasing age, DM prevalence increased gradually. Nearly two-fifths of hypertensive individuals between 60 and 69 years of age suffered from DM, and the situation was worse between 70 to 79 years, more than two-fifths of hypertensive individuals suffered from DM. With increasing BMI, DM prevalence also tended to increase gradually.

As shown above, a previous DM diagnosis rate of 11.2% was obtained in hypertensive populations aged 40 to 79 years; undiagnosed rate was 20.8% in this study. It means that people with hypertension aged 40 to 79 years in Southwest China’s community, 1/5 of them have underlying DM.

It was discovered that newly diagnosed DM cases accounted for 65.1% of all DM patients, this proportion was higher than that in Spain outpatient hypertensive patients (45.3%) [14], and even more higher than that in Chinese outpatient hypertensive patients (34.7%) [15]. Our findings indicate that most diabetic patients of community were not timely recognized, especially the relatively young patients. Compared with outpatients, the prevalence of unrecognized DM in hypertensive patients may be higher. It is very important to enforce DM screening in Chinese hypertension patients of broad community.

In the general Chinese population, 46.6% of newly diagnosed DM have isolated increased 2-hPG levels [3]. An investigation with 499 new-onset hypertensions in Chinese Han outpatients found that 57.6% of newly diagnosed DM have increased 2-hpG levels [16], suggesting that an important portion of newly diagnosed DM cases depend on 2-hpG levels. The Rancho Bernardo Study reported that 60% patients with DM were identified by 2-hPG tests among individuals aged > 50 years [17]. The above studies showed that nearly half (or even more) of individuals would be diagnosed while relying on 2-hpG levels. The current study found that relying solely on fasting glucose yielded 35% of new DM diagnoses. Meanwhile, 65% of newly diagnosed DM were detected based on 2-hPG levels, indicating that nearly two-thirds of new DM diagnoses rely on 2-hpG among hypertensive patients aged 40 to 79 years. At present, some Chinese urban community health service institutions routinely check FPG for middle-aged and elderly people, with postprandial blood glucose often overlooked. Although some DM cases are detected this way, it may yield a high proportion of undiagnosed DM. We found that most new DM cases would be missed if only fasting blood glucose test is performed in hypertensive patients, and 2-hpG level detection for DM diagnosis may have a better value compared with fasting glucose. Chinese doctors often assess hypertensive outpatients only for fasting glucose levels, missing an important fraction of diabetic patients. We recommend that hypertensive patients aged 40 to 79 years should regularly submit to OGTT for timely detection of DM. The current study in communities showed that more than one-third of hypertensive patients aged 60 years and above suffer from DM. Therefore, elderly people with hypertension should be the focus of screening. With the rapid economic and social development, China has undergone a speedy urbanization in recent years.

Chinese adults now have a significantly higher obesity prevalence [18]. Overweight and obesity in the U.S. adult population are closely related to the prevalence of DM [19]. The current findings suggest that DM prevalence is significantly higher in hypertensive patients with overweight or obesity aged 40 to 79 years. Therefore, prevention of overweight, obesity, central obesity, and other metabolic disorders in Chinese population may reduce or delay the occurrence or development of DM in hypertension patients.

The present study has some limitations. First, HbA1c is more accurate for diabetes diagnostic, and we did not measure this index due to the large sample size and funding limitation. This limitation may underestimate or overestimate the prevalence of diabetes in this study population. Second, some comorbidities and medical history data are unavailable that could have some influences on the prevalence of DM both diagnosed and undiagnosed. Several diseases were found to be associated with a greater prevalence of both diagnosed and undiagnosed diabetes, such as PCOS [20–22]. Other diseases, for converse, are characterized by a reduced prevalence of diabetes [23,24]. Third, no information is provided for current or past treatment, diabetogenic medications such as corticosteroids and protective medications such as biologics. Both of them could affect the prevalence of diagnosed and undiagnosed diabetes [25–27]. Finally, the results of the study can only represent the population of the southwest region. This point should be cautious in other population setting.

In summary, in hypertensive patients aged 40 to 79 years in Southwest China, the prevalence of diabetes was high, And the more serious is that the prevalence of unrecognized diabetes was also high. Approximately one-in-three hypertensive patients aged 40 to 79 years are diabetics, one-in-five of hypertensive patients aged 40 to 79 years have underlying diabetes. Findings indicate that most diabetic patients of community were not timely recognized.

## Supporting information

S1 Data Data of epidemic status of diabetes and related cardiovascular risk factors. (XLSX)

S1 Questionnaire Epidemic status of diabetes and related cardiovascular risk factors. (DOC)
