# Supplementary material for: Prevalence of diabetes and unrecognized diabetes in hypertensive patients aged 40 to 79 years in southwest China
Source: PLoS One. 2017 Feb 13;12(2):e0170250. doi: 10.1371/journal.pone.0170250 (PMC5305248; doi:10.1371/journal.pone.0170250)
Supplement: S1 Questionnaire — (DOC) [file pone.0170250.s002.doc]

糖尿病及相关心血管危险因素流行情况调查表

num 编号 ######

一般资料

a2 性别 # 1.男 2.女

a32 出生年月 <yyyy/mm/dd>

a5 文化程度 # 1.文盲 2.小学 3.初中4.高中，技校 5.中专 6.大专 7.本科及以上

a6 婚姻 # 1.已婚 2.未婚 3.离婚 4.丧偶

医保情况

a71 无医保 <y>

a72 职工医疗保险 <y>

a73 公费医疗社保 <y>

a74 商业保险 <y>

a75 新型农村合作医疗 <y>

a76 贫困救助 <y>

a77 劳保医疗 <y>

a78 其它 ______________________________

去年你家庭人均月收入 # 1、小于1000元 2、1000~元 3、2000~元 4、3000~元 5、5000及以上元

生活方式

吸烟情况

b11 是否吸烟 # 1、吸烟 2、不吸烟 3、已戒烟

b12 吸烟年数 ##

b13 每日吸烟量（支数） ###

b14 戒烟年数 ##

饮酒情况

b21 是否饮酒 # 1、饮酒 2、不饮酒 3、已戒酒

b22 饮酒年数 ##

b23 每日饮酒量 ##

b24 戒酒年数 ##

b3 你工作体力强度是 # 1.非常轻-坐着 2.轻-站立或来回走动、驾车、家务 3.中-轻工农业 一般室外内活动

庭院操作、搬运或举起轻物等 4.重-重工农业、室外建筑 、野外施工、搬运或举起重物等、剧烈体育活动等

b4 近一年来你体育锻炼频率 # 1.不锻炼 2. 每周小于3次 3.每周大于或等于3次 4.无规律

b5 每次锻炼时间 # 1.小于30分钟 2.30分钟-1小时 3.大于1小时

饮食习惯

b61 摄盐 # 1. 偏咸 2. 适中 3. 偏淡

b62 油腻食物 # 1. 偏油 2. 适中 3. 偏淡

b63 近一月来你平均每日主食量 # 1.小于150g 2. 150-300g 3.300g-450g . 4.大于450g

个人史

生育情况

c11 妊娠次数 #

c12 分娩次数 #

c13 是否绝经 # 1、绝经 2、未绝经

c14 绝经年数 ##

c2 你分娩的最重婴儿体重 # 1、小于4000g 2、大于4000g 3、不清楚

c3 你妊娠时诊断过妊娠高血压吗 # 1.有 2.没有 3.不清楚

c4 你妊娠时诊断过妊娠糖尿病吗 # 1.有 2.没有 3.不清楚

病 史

高血压史:

d1 你最近一年测过血压吗 # 1.测过 2.未测过

d2 你有高血压病吗 # 1.有 2.无 3.不清楚

d3 首次诊断在多少年前 ##

最高血压

d41 收缩压 ###

d42 舒张压 ###

d5 治疗情况 # 1. 规则治疗2.间断治疗 3. 未治疗

冠心病史：

d61 你有冠心病吗 # 1.有 2.无 3.不清楚

d62 患冠心病年数 ##

脑卒中及老年痴呆史：

d71 是否有中风 # 1、有 2、无 3、不清楚

d72 第一次是发生在多少年前 ##

d73 总共发生多少次 ##

d8 中风类型 # 1、出血 2、梗塞 3、不清楚

肾脏病史：

d91 是否有肾脏病史 # 1.有 2. 无 3.不清楚

d92 肾脏病史多少年 ##

d101 慢性肾脏病类型 # 1.慢性肾小球肾炎 2.慢性肾盂肾炎 3.糖尿病肾病

其它类型 ______________________________9不清楚

糖尿病史：

d11 测过血糖吗 # 1.测过2.未测过

d121 是否有糖尿病史 # 1.有 2. 无 3.不清楚

d122 糖尿病史多少年 ##

d13 诊断医院 # 1、三级医院 2、二级医院 3、社区医疗服务中心

d14 诊断类型 # 1. 1型 2. 2型 3.糖尿病前期 4.不清楚

d15 治疗情况 # 1. 规则治疗 2.间断治疗 3. 未治疗

d161 现在使用哪些降糖药物 # 1.胰岛素：2.磺脲类：3.双胍类： 4.2-糖苷酶抑制剂5.胰岛素增敏剂

6.胰岛素促泌剂：7.中药 8.其他

d162 其它药物 ______________________________

d171 你最近一年测过HbA1c吗 # 1.测过 2.未测过

d172 测的结果为 ##.#%

d18 最近一次空腹血糖 ##.#mmol/L

d19 最近一次餐后血糖 ##.#mmol/L

d20 糖尿病并发症 # 1. 糖尿病肾病 2. 视网膜病变 3. 糖尿病神经病变 4. 糖尿病足 5. 视力 6.听力明显障碍

d21 高尿酸血症（痛风） # 1.有 2.无 3.不清楚

血脂紊乱史：

d22 有无血脂紊乱 # 1.有 2.无 3.不清楚

d23 类型 # 1.高甘油三酯 2.高胆固醇 3.混合型 4.不清楚

d24 治疗情况 # 1. 规则治疗 2.间断治疗 3.未治疗

d251 其它病史 # 1、无 2、心脏疾病 3、COPD 4、肿瘤 5、其他

d252 其它 ___________________________

家族史

1）父亲患病情况

d261 糖尿病 # 1.有 2. 无 3.不清楚

d262 高血压 # 1.有 2. 无 3.不清楚

d263 脑卒中 # 1.有 2. 无 3.不清楚

d264 冠心病（如心梗） # 1.有 2. 无 3.不清楚

d265 高血脂 # 1.有 2. 无 3.不清楚

2）母亲患病情况

d271 糖尿病 # 1.有 2. 无 3.不清楚

d272 高血压 # 1.有 2. 无 3.不清楚

d273 脑卒中 # 1.有 2. 无 3.不清楚

d274 冠心病（如心梗） # 1.有 2. 无 3.不清楚

d275 高血脂 # 1.有 2. 无 3.不清楚

3）兄弟姐妹患病情况

d281 糖尿病 # 1.有 2. 无 3.不清楚

d282 高血压 # 1.有 2. 无 3.不清楚

d283 脑卒中 # 1.有 2. 无 3.不清楚

d284 冠心病（如心梗） # 1.有 2. 无 3.不清楚

d285 高血脂 # 1.有 2. 无 3.不清楚

查体

e1 一般情况 # 1. 良好 2. 一般 3. 较差

e2 身高 ###cm

e3 体重 ###kg

e4 腰围 ###cm

e5 臀围 ###cm

血压坐位一次

e6 收缩压 ###mmHg

e7 舒张压 ###mmHg

血压坐位二次

e8 收缩压 ###mmHg

e9 舒张压 ###mmHg

e10 心率 ###次/分

e11 心律 # 1、齐 2、早搏 3、房颤 4、阵发过速 5、过缓 6、其它异常

e12 其它异常______________________________

辅助检查

f1 空腹血糖 ##.#mmol/l f2餐后2小时 ##.#mmol/l

f3 甘油三酯 #.##mmol/l f4 胆固醇 #.##mmol/l

f5HDL #.##mmol/l f6 LDL #.##mmol/l

f7 尿酸 ###umol/l
